# Development and validation of nomograms for predicting prognosis in patients with resectable bladder urothelial carcinoma undergoing radical cystectomy: a multicenter retrospective study

**DOI:** 10.3389/fonc.2025.1571604

**Published:** 2025-07-03

**Authors:** Junjie Ji, Zengjin Wen, Yu Yao, Lei Jiang, Qingya Yang, Guiming Zhang

**Affiliations:** ^1^ Department of Urology, The Affiliated Hospital of Qingdao University, Qingdao, China; ^2^ School of Rehabilitation, Capital Medical University, Beijing, China; ^3^ Department of Urology, China Rehabilitation Research Center, Beijing, China; ^4^ Department of Thoracic Surgery II, Key Laboratory of Carcinogenesis and Translational Research (Ministry of Education), Peking University Cancer Hospital and Institute, Beijing, China; ^5^ Department of Urology, Qilu Hospital (Qingdao), Cheeloo College of Medicine, Shandong University, Qingdao, China

**Keywords:** bladder cancer, bladder urothelial carcinoma, radical cystectomy, prognosis, nomogram

## Abstract

**Purpose:**

This study aimed to construct and validate nomograms for the prediction of overall survival (OS), cancer-specific survival (CSS), and disease-free survival (DFS) in patients with resectable bladder urothelial carcinoma (BUC) after radical cystectomy (RC).

**Methods:**

We retrospectively collected the demographic, pathological, imaging, and laboratory data from patients with BUC who underwent RC. The training cohort included patients from the Affiliated Hospital of Qingdao University from January 2018 to December 2021, while the test cohort included patients from the same hospital between January 2016 and December 2017, along with patients from Qilu Hospital of Shandong University. Univariate and multivariate Cox regression analyses were conducted to identify independent predictors of OS, CSS, and DFS. The performance of the nomograms was evaluated using Harrell’s concordance index (*C*-index), the area under the receiver operating characteristic (ROC) curve (AUC), the corrected AUC following 1,000 bootstrap resamplings with calibration curves, and decision curve analysis in both cohort validations.

**Results:**

A total of 393 patients were included in the training cohort, while 156 patients comprised the test cohort. Multivariate analyses revealed that age, tumor size, lymph node metastasis (LNM), lymphovascular invasion (LVI), urea nitrogen, creatinine, and the albumin/fibrinogen ratio (AFR) were independent predictors for OS. For CSS, the independent predictors were tumor size, LNM, LVI, urea nitrogen, and AFR. LNM and LVI were the independent predictors for DFS. The nomograms for OS and CSS demonstrated high predictive accuracy with robust *C*C-indexes and ROC curves, along with reliable calibration curves with corrected AUCs and clinical utility in both cohorts. The DFS nomogram also showed high predictive accuracy with stable corrected AUCs in both cohorts.

**Conclusion:**

We constructed OS, CSS, and DFS nomograms to predict prognosis in patients with BUC treated with RC. These nomograms exhibited high accuracy, reliability, and clinical utility in predicting outcomes in both cohorts.

## 1 Introduction

Bladder cancer accounts for 6% of new estimated cancers and 4% of cancer-related deaths and is the fourth most common cancer in men in recent years (1–3). Bladder cancer at the initial diagnosis can mainly be categorized as non-muscle-invasive bladder cancer (NMIBC) or muscle-invasive bladder cancer (MIBC), whose pathological type is primarily bladder urothelial carcinoma (BUC) (4, 5). Radical cystectomy (RC) with bilateral pelvic lymph node dissection and urinary diversion remains the standard treatment for patients with resectable MIBC (T2-4a, N0/1-x, M0) and high-risk NMIBC (6). NMIBC is easily recurrent and progressive to MIBC (7–10), and patients with MIBC have a poor prognosis, with an overall 5-year survival rate of 40%–50% after RC but less than 15% if untreated (10–12). Therefore, there is an urgent need for a distinct risk prediction model for the prognosis of patients with BUC after RC for effective monitoring and timely intervention.

A reliable multivariate prediction model, in particular a visual nomogram, could facilitate the accurate quantification of the mortality risk of patients with BUC, enabling clinicians to quickly identify groups with poor prognoses and refer individuals to psychiatrists for timely interventions in order to help patients with BUC after RC, ultimately improving prognoses as much as possible (13–15). Several studies have focused on the prediction of lymph node metastasis (LNM), distant metastasis, and prognosis in patients with BUC using nomograms (16–19). However, studies comprehensively predicting the prognosis of these patients after RC using multidimensional variables from two real-world institutions are scarce. Therefore, this retrospective study aimed to use the demographic, pathological, radiologic, and laboratory data from two institutions to identify potential independent risk factors and to establish and validate prognostic nomograms for predicting the overall survival (OS), cancer-specific survival (CSS), and disease-free survival (DFS) in patients with resectable BUC undergoing RC.

## 2 Materials and methods

### 2.1 Patient selection

This study was approved by the Medical Ethics Committee of the Affiliated Hospital of Qingdao University (No QDFYWZLL29357) and was carried out following the Declaration of Helsinki of the World Medical Association. We retrospectively enrolled patients with bladder cancer who received RC and bilateral lymphadenectomy at the Affiliated Hospital of Qingdao University from January 2016 to December 2021 and at the Qingdao Campus of Qilu Hospital of Shandong University from January 2017 to December 2022. Patients from the Affiliated Hospital of Qingdao University between January 2018 and December 2021 were assigned to the training cohort, while those from the Affiliated Hospital of Qingdao University from January 2016 to December 2017 and those from Qilu Hospital of Shandong University were assigned to the test cohort.

Patients were excluded based on the following criteria: a) age <18 years; b) incomplete imaging examination data before RC; c) incomplete laboratory measurements 1 month before RC; d) tumor originating from other sites other than the bladder; e) severe or end-stage chronic kidney disease or severe inflammation; f) pathological diagnosis of non-urothelial carcinoma; and g) lack of follow-up information for calculating any of the survival endpoints.

### 2.2 Characteristic collection and endpoints

The following clinical characteristics of the included cases were collected: a) demographic data, including age, sex, and body mass index (BMI); b) pathologic data from RC, including grade, papillary, urothelial variants, T stage, margin, tumor size, LNM, nerve infiltration, and lymphovascular invasion (LVI); c) imaging data before RC, including hydronephrosis; and d) laboratory measurements before RC, including the hemoglobin, urea nitrogen, creatinine, neutrophil count, lymphocyte count, platelet count, monocyte count, alanine transaminase, aspartate transaminase, albumin, and fibrinogen.

In addition, new laboratory ratios were calculated using some of these measurements. The respective cell counts were used to calculate the neutrophil-to-lymphocyte ratio (NLR), the platelet-to-lymphocyte ratio (PLR), the monocyte-to-lymphocyte ratio (MLR), and the neutrophil-to-platelet ratio (NPR). The systemic immune-inflammation index (SII) was defined by multiplying the platelet count by the neutrophil count and then dividing this value by the lymphocyte count. The aspartate transaminase-to-alanine transaminase ratio is known as the De Ritis ratio (DRR). Finally, the albumin-to-fibrinogen ratio (AFR) was also calculated and defined. In order to reduce the impact of surgery on the baseline laboratory measurements, the laboratory characteristics were collected before transurethral resection of bladder tumor (TURBT) when TURBT was performed within 1 month before RC. Otherwise, these data were collected before RC rather than TURBT.

The primary endpoint was OS, which was defined as the time from the completion of RC to death from any cause. CSS and DFS were set as the secondary endpoints and were defined as the time from the completion of RC to death from BUC and the time from the completion of RC to clinical tumor recurrence or death from any cause, respectively.

### 2.3 Conversion and selection of independent risk variables

The median values of the continuous variables, including age, BMI, laboratory measurements, and laboratory ratios for the analysis of OS in the training cohort, were calculated and used as cutoff points to convert all corresponding continuous variables for the analyses of OS, CSS, and DFS in both cohorts into binary categorical variables. Univariate Cox regression analyses were performed on the training cohort to identify potential risk factors for OS, CSS, and DFS. Significant variables from the univariate analyses were included in the multivariate Cox regression analyses to identify independent predictors of OS, CSS, and DFS in patients with BUC treated with RC.

### 2.4 Establishment and validation of the nomograms

The significant risk factors identified from the multivariate Cox regression analyses in the training cohort were used to construct an OS nomogram for prediction of the 2-, 3-, and 5-year OS rates. The CSS and DFS nomograms were also constructed from the multivariate Cox regression analyses to predict the corresponding survival rates. Various significant risk factors were directly associated with the nomogram scores, and the summation of the scores, i.e., the total points, could be applied to predict the prognosis of patients with BUC undergoing RC. To quantify the discrimination performance of the nomograms, we measured Harrell’s concordance index (*C*-index), plotted the receiver operating characteristic (ROC) curves associated with the corresponding nomograms, and calculated the area under the ROC curves (AUC) to assess the accuracy of the nomograms. Next, we used 1,000 bootstrap resampling validations and calculated the relatively corrected *C*-index and corrected AUCs. Calibration curves and comparison with the primary AUCs were applied to evaluate the calibration of the nomograms. Decision curve analyses (DCA) were performed to assess the application value of the nomograms in predicting survival rates by showing potential clinical benefits at each risk threshold level.

Subsequently, we applied the nomograms constructed from the training cohort to the test cohort for further validation. *C*-indexes, ROC curves with AUCs, 1,000 bootstrap resampling validations with corrected AUCs, and DCA curves were also used to assess the prediction accuracy, stability, and clinical application value of the nomogram models in the training cohort.

### 2.5 Statistical analyses

Continuous variables were converted to binary categorical variables using the median calculated from the data of the OS analysis in the training cohort. Categorical variables are presented as frequencies and proportions. Chi-squared tests or Fisher’s exact tests were used for comparison of the variables between the training and test cohorts. Univariate Cox regression analyses were performed on all parameters in the training cohort to identify potential risk factors for OS, CSS, and DFS. The statistically significant variables from the univariate Cox regression analyses were included in the multivariate Cox regression analyses. Multivariate Cox regression analyses of the forward likelihood ratio (LR) methods were applied to calculate the hazard ratio (HR) with 95% confidence interval (CI) and identify independent risk factors for OS, CSS, and DFS. The R software packages, including “rms,” “pROC,” “plotROC,” “riskRegression,” “dcurves,” “survival,” “ggplot2,” and “dplyr”, were used to calculate the C-indexes, ROC curves, bootstrap resampling validations with calibration curves, and DCA curves. A two-sided *p* < 0.05 was considered a measure of statistical significance. All statistical analyses were performed using SPSS software (version 24.0) and R software (version 4.1.0).

## 3 Results

### 3.1 Clinical characteristics

**Figure 1:**
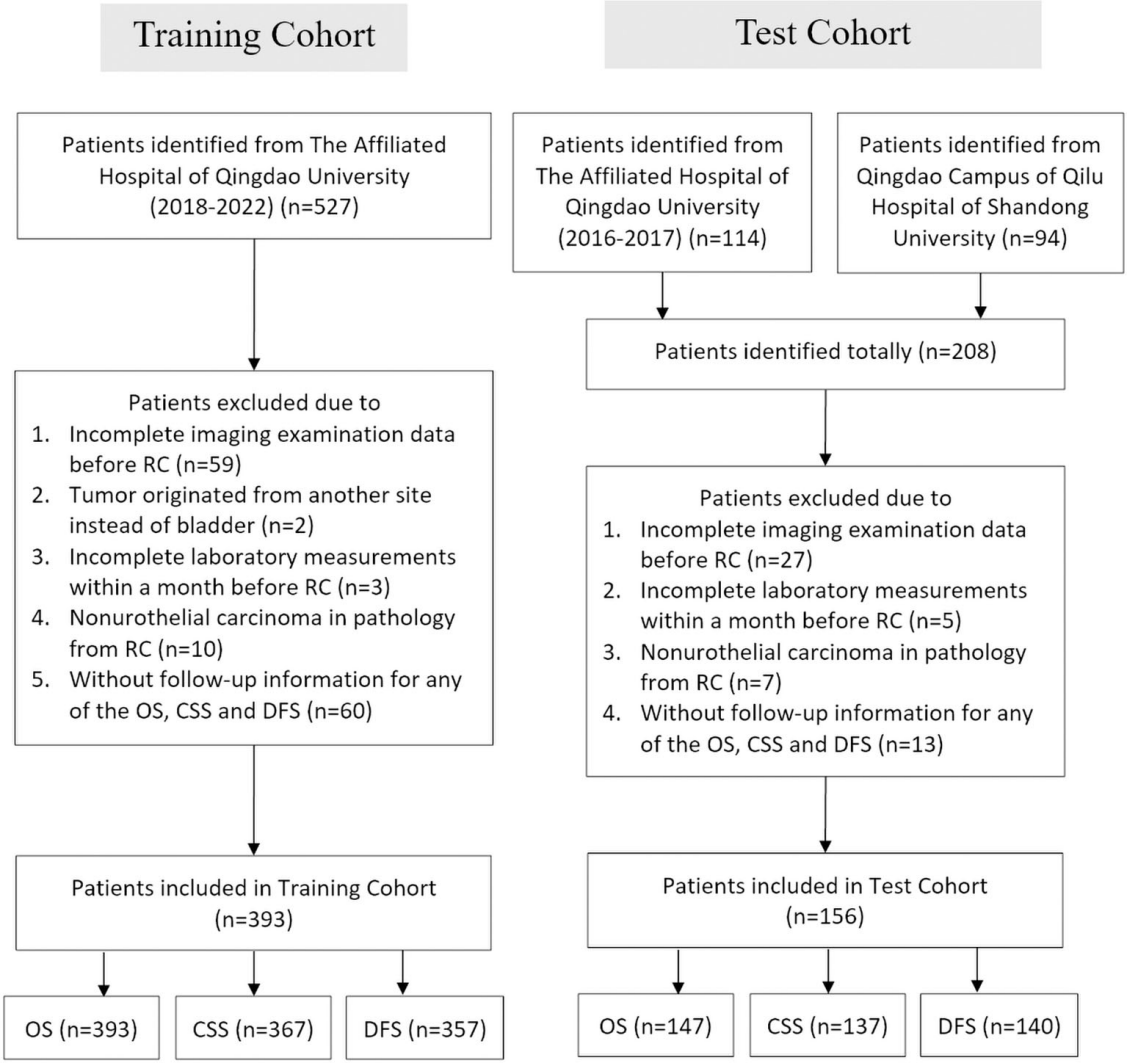
Flowchart of the patient selection process in the training and test cohorts. *RC*, radical cystectomy; *OS*, overall survival; *CSS*, cancer-specific survival; *DFS*, disease-free survival.

A total of 641 patients from the Affiliated Hospital of Qingdao University and 94 patients from the Qingdao Campus of the Qilu Hospital of Shandong University were enrolled in this study. Following application of the specified criteria, a total of 549 patients were selected for analysis, 126 of whom experienced mortality during the follow-up period. The median OS, CSS, and DFS were not achieved within the study time frame. However, the upper quartile OS, CSS, and DFS were 46, 64, and 45 months, respectively. There were 393 patients from the Affiliated Hospital of Qingdao University between January 2018 and December 2021 included in the training cohort (393 cases for OS analysis, 367 cases for CSS analysis, and 357 cases for DFS analysis), while patients from the Affiliated Hospital of Qingdao University from January 2016 to December 2017 and from Qilu Hospital of Shandong University were included in the test cohort (156 in total; 147 cases for OS analysis, 137 cases for CSS analysis, and 140 cases for DFS analysis) (**Figure 1**).

Comparisons of the baseline characteristics between the training cohort and the test cohort for the analysis of OS, CSS, and DFS are shown in **Table 1** and **Supplementary Tables S1**, **S2** using chi-squared tests or Fisher’s exact tests. The results showed that most variables did not differ significantly between the training cohort and the test cohort. Only the T stage in the CSS analysis, the margin, PLR, and SII in all analyses, and the NPR in both CSS and DFS analyses were significantly different between the two cohorts. Therefore, the clinical baseline characteristics between the two cohorts were similarly distributed.

**Table 1:** Comparison of the baseline characteristics between the two cohorts for overall survival (OS).

Characteristics		Training cohort (*n* = 393)	Test cohort (*n* = 147)	*p*-value
Demography				
Age (years)	≤66	199 (50.6%)	77 (52.4%)	0.718
	>66	194 (49.4%)	70 (47.6%)	
Sex	Men	338 (86.0%)	126 (85.7%)	0.931
	Women	55 (14.0%)	21 (14.3%)	
BMI (kg/m^2^)	≤23.9	204 (51.9%)	71 (48.3%)	0.455
	>23.9	189 (48.1%)	76 (51.7%)	
Pathology				
Grade	High	349 (88.8%)	128 (87.1%)	0.577
	Low	44 (11.2%)	19 (12.9%)	
Papillary	Yes	161 (41.0%)	73 (49.7%)	0.070
	No	232 (59.0%)	74 (50.3%)	
Urothelial variants	Yes	80 (20.4%)	23 (15.6%)	0.215
	No	313 (79.6%)	124 (84.4%)	
T stage	T1	149 (37.9%)	47 (32.0%)	0.065
	Ta	13 (3.3%)	4 (2.7%)	
	Tis	4 (1.0%)	2 (1.4%)	
	T2	120 (30.5%)	45 (30.6%)	
	T3	82 (20.9%)	27 (18.3%)	
	T4	25 (6.4%)	22 (15.0%)	
Margin	Positive	10 (2.5%)	12 (8.2%)	0.003^**^
	Negative	383 (97.5%)	135 (91.8%)	
Tumor size (cm)	≥4	159 (40.5%)	61 (41.5%)	0.827
	<4	234 (59.5%)	86 (58.5%)	
LNM	Yes	61 (15.5%)	26 (17.7%)	0.542
	No	332 (84.5%)	121 (82.3%)	
Nerve infiltration	Yes	70 (17.8%)	29 (19.7%)	0.609
	No	323 (82.2%)	118 (80.3%)	
LVI	Yes	114 (29.0%)	41 (27.9%)	0.799
	No	279 (71.0%)	106 (72.1%)	
Imaging				
Hydronephrosis	Yes	98 (24.9%)	41 (27.9%)	0.485
	No	295 (75.1%)	106 (72.1%)	
Laboratory				
Hemoglobin	≤139	208 (52.9%)	79 (53.7%)	0.866
	>139	185 (47.1%)	68 (46.3%)	
Urea nitrogen	≤6.34	197 (50.1%)	79 (53.7%)	0.455
	>6.34	196 (49.9%)	68 (46.3%)	
Creatinine	≤79	199 (50.6%)	75 (51.0%)	0.937
	>79	194 (49.4%)	72 (49.9%)	
NLR	≤2.19	197 (50.1%)	83 (56.5%)	0.190
	>2.19	196 (49.9%)	64 (43.5%)	
PLR	≤130.29	197 (50.1%)	89 (60.5%)	0.031^*^
	>130.29	196 (49.9%)	58 (39.5%)	
MLR	≤0.27	197 (50.1%)	81 (55.1%)	0.303
	>0.27	196 (49.9%)	66 (44.9%)	
NPR	≤0.018	197 (50.1%)	87 (59.2%)	0.061
	>0.018	196 (49.9%)	60 (40.8%)	
SII	≤524.95	197 (50.1%)	91 (61.9%)	0.015*
	>524.95	196 (49.9%)	56 (38.1%)	
DRR	≤1.05	198 (50.4%)	71 (48.3%)	0.667
	>1.05	195 (49.6%)	76 (51.7%)	
AFR	≤13.24	197 (50.1%)	75 (51.0%)	0.853
	>13.24	196 (49.9%)	72 (49.0%)	

*BMI*, body mass index; *LNM*, lymph node metastasis.

*p < 0.05; **p < 0.01; ***p < 0.001.

*LVI*, lymphovascular invasion; *NLR*, neutrophil-to-lymphocyte ratio; *PLR*, platelet-to-lymphocyte ratio; *MLR*, monocyte-to-lymphocyte ratio; *NPR*, neutrophil-to-platelet ratio; *SII*, systemic immune-inflammation index; *DRR*, De Ritis ratio; *AFR*, albumin-to-fibrinogen ratio.

*p < 0.05; **p < 0.01; ***p < 0.001.

### 3.2 Independent predictors for prognosis and model construction

**Figure 2:**
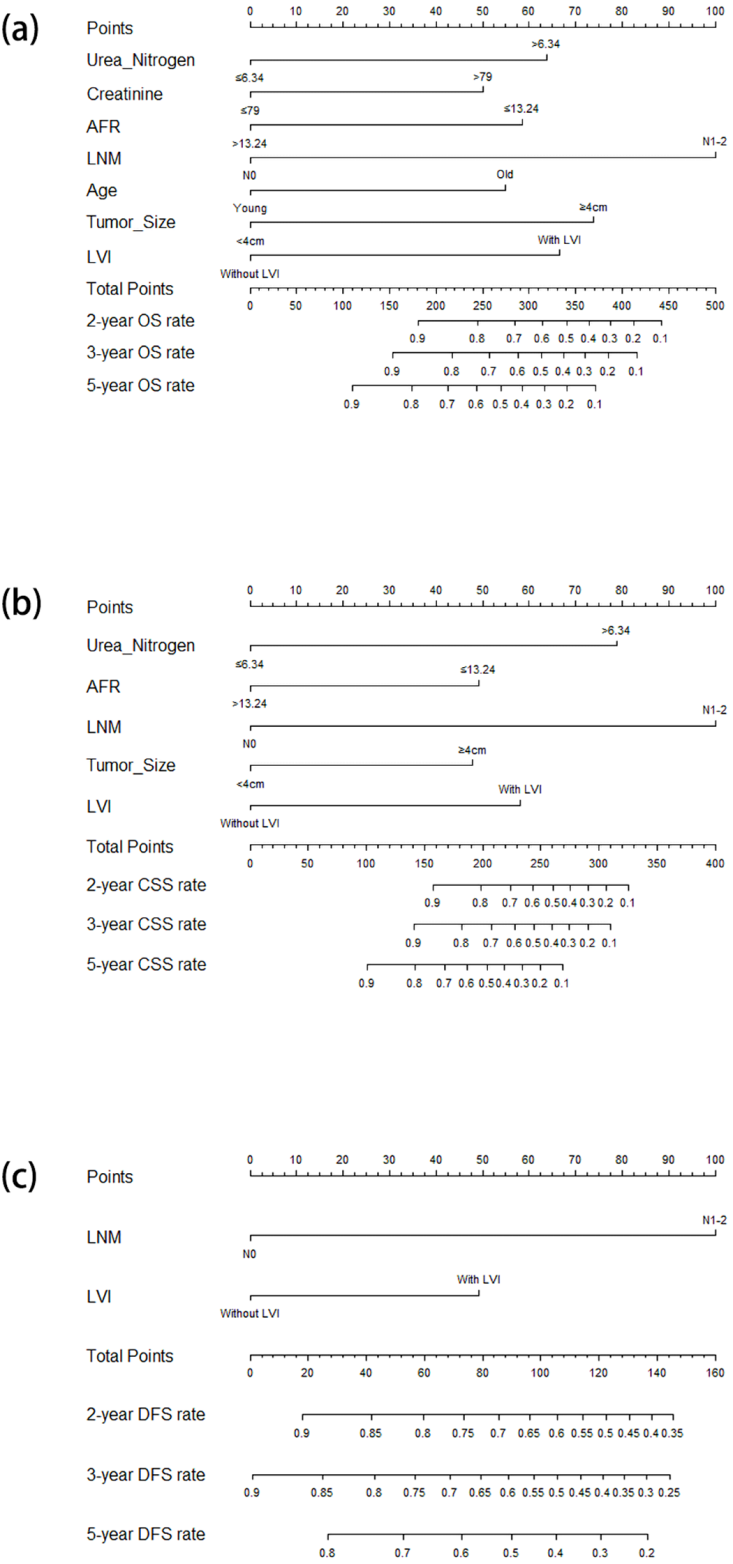
Nomograms for predicting the OS **(a)**, CSS **(b)**, and DFS **(c)** in patients with resectable BUC receiving RC. *OS*, overall survival; *CSS*, cancer-specific survival; *DFS*, disease-free survival; *BUC*, bladder urothelial carcinoma; *RC*, radical cystectomy.

In the univariate Cox regression analysis for OS, age, grade, urothelial variants, T stage, margin, tumor size, LNM, nerve infiltration, LVI, hydronephrosis, hemoglobin, urea nitrogen, creatinine, NLR, MLR, SII, and AFR were identified as significant variables, listed in the univariate analysis section in **Table 2**. After multivariate Cox regression analysis, older age (HR = 1.904, 95%CI = 1.193–3.040, *p* = 0.007), larger tumor size (HR = 2.358, 95%CI = 1.483–3.749, *p* < 0.001), LNM (HR = 3.197, 95%CI = 1.855–5.509, *p* < 0.001), LVI (HR = 2.190, 95%CI = 1.293–3.709, *p* = 0.004), higher serum urea nitrogen (HR = 2.096, 95%CI = 1.304–3.370, *p* = 0.002), higher serum creatinine (HR = 1.792, 95%CI = 1.100–2.918, *p* = 0.019), and lower AFR (HR = 0.504, 95%CI = 0.299–0.850, *p* = 0.010) were found to be independently associated with poor OS (**Table 2**). The nomogram for predicting OS is shown in **Figure 2a**.

**Table 2:** Univariate and multivariate Cox regression analyses for prediction of overall survival (OS) in the training cohort (forward LR).

Indicators	Univariate analysis		Multivariate analysis	
	HR (95%CI)	*p*-value	HR (95%CI)	*p*-value
Age (years)				
≤66	Reference		Reference	
>66	2.035 (1.289–3.213)	0.002^**^	1.904 (1.193–3.040)	0.007^**^
Grade				
Low	Reference			
High	2.837 (1.037–7.759)	0.042^*^		
Urothelial variants				
No	Reference			
Yes	2.089 (1.293–3.374)	0.003^**^		
T stage				
T1, Ta, Tis	Reference			
T2	1.713 (0.897–3.269)	0.103		
T3	4.826 (2.677–8.698)	<0.001^***^		
T4	6.371 (2.972–13.661)	<0.001^***^		
Margin				
Negative	Reference			
Positive	4.322 (1.741–10.728)	0.002^**^		
Tumor size (cm)				
<4	Reference		Reference	
≥4	2.590 (1.651–4.061)	<0.001^***^	2.358 (1.483–3.749)	<0.001^***^
LNM				
No	Reference		Reference	
Yes	5.979 (3.820–9.358)	<0.001^***^	3.197 (1.855–5.509)	<0.001^***^
Nerve infiltration				
No	Reference			
Yes	3.560 (2.260–5.608)	<0.001^***^		
LVI				
No	Reference		Reference	
Yes	4.159 (2.665–6.489)	<0.001^***^	2.190 (1.293–3.709)	0.004^**^
Hydronephrosis				
No	Reference			
Yes	2.688 (1.723–4.193)	<0.001^***^		
Hemoglobin				
≤139	Reference			
>139	0.487 (0.305–0.777)	0.003^**^		
Urea nitrogen				
≤6.34	Reference		Reference	
>6.34	2.023 (1.277–3.204)	0.003^**^	2.096 (1.304–3.370)	0.002^**^
Creatinine				
≤79	Reference		Reference	
>79	2.361 (1.483–3.758)	<0.001^***^	1.792 (1.100–2.918)	0.019^*^
NLR				
≤2.19	Reference			
>2.19	1.884 (1.197–2.965)	0.006^**^		
MLR				
≤0.27	Reference			
>0.27	2.529 (1.581–4.046)	<0.001^***^		
SII				
≤524.95	Reference			
>524.95	1.668 (1.066–2.610)	0.025^*^		
AFR				
≤13.24	Reference		Reference	
>13.24	0.310 (0.188–0.510)	<0.001^***^	0.504 (0.299–0.850)	0.010^**^

*HR*, hazard ratio; *CI*, confidence interval; *LNM*, lymph node metastasis; *LVI*, lymphovascular invasion.

*p < 0.05; **p < 0.01; ***p < 0.001.

*HR*, hazard ratio; *CI*, confidence interval; *NLR*, neutrophil-to-lymphocyte ratio; *MLR*, monocyte-to-lymphocyte ratio; *SII*, systemic immune-inflammation index; *AFR*, albumin-to-fibrinogen ratio.

*p<0.05; **p<0.01; ***p<0.001.

In the univariate Cox regression analysis for CSS, urothelial variants, T stage, margin, tumor size, LNM, nerve infiltration, LVI, hydronephrosis, urea nitrogen, creatinine, MLR, SII, and AFR were identified as significant factors, listed in the univariate analysis section in **Table 3**. The multivariate Cox regression analysis demonstrated that tumor size (HR = 2.386, 95%CI = 1.361–4.184, *p* = 0.002), LNM (HR = 6.115, 95%CI = 3.168–11.804, *p* < 0.001), LVI (HR = 2.886, 95%CI = 1.479–5.629, *p* = 0.002), serum urea nitrogen (HR = 4.169, 95%CI = 2.276–7.636, *p* < 0.001), and AFR (HR = 0.410, 95%CI = 0.211–0.796, *p* = 0.008) were independent risk factors for CSS (**Table 3**). The nomogram for predicting CSS is shown in **Figure 2b**.

**Table 3:** Univariate and multivariate Cox regression analyses for the prediction of CSS in the training cohort (forward LR).

Indicators	Univariate analysis		Multivariate analysis	
	HR (95%CI)	*p*-value	HR (95%CI)	*p*-value
Urothelial variants				
No	Reference			
Yes	1.916 (1.054–3.482)	0.033^*^		
T stage				
T1, Ta, Tis	Reference			
T2	2.017 (0.811–5.015)	0.131		
T3	8.458 (3.826–18.700)	<0.001^***^		
T4	12.244 (4.692–31.951)	<0.001^***^		
Margin				
Negative	Reference			
Positive	4.073 (1.266–13.104)	0.018^*^		
Tumor size (cm)				
<4	Reference		Reference	
≥4	2.627 (1.520–4.542)	<0.001^***^	2.386 (1.361–4.184)	0.002^**^
LNM				
No	Reference		Reference	
Yes	9.981 (5.810–17.147)	<0.001^***^	6.115 (3.168–11.804)	<0.001^***^
Nerve infiltration				
No	Reference			
Yes	4.940 (2.883–8.467)	<0.001^***^		
LVI				
No	Reference		Reference	
Yes	5.968 (3.408–10.450)	<0.001^***^	2.886 (1.479–5.629)	0.002^**^
Hydronephrosis				
No	Reference			
Yes	2.996 (1.749–5.131)	<0.001^***^		
Urea nitrogen				
≤6.34	Reference		Reference	
>6.34	2.686 (1.497–4.819)	0.001^**^	4.169 (2.276–7.636)	<0.001^***^
Creatinine				
≤79	Reference			
>79	2.159 (1.240–3.756)	0.006^**^		
MLR				
≤0.27	Reference			
>0.27	2.551 (1.445–4.506)	0.001^**^		
SII				
≤524.95	Reference			
>524.95	1.778 (1.028–3.076)	0.040^*^		
AFR				
≤13.24	Reference		Reference	
>13.24	0.240 (0.127–0.457)	<0.001^***^	0.410 (0.211–0.796)	0.008^**^

*HR*, hazard ratio; *CI*, confidence interval; *LNM*, lymph node metastasis; *LVI*, lymphovascular invasion.

*p < 0.05; **p < 0.01; ***p < 0.001.

*HR*, hazard ratio; *CI*, confidence interval; *MLR*, monocyte-to-lymphocyte ratio; *SII*, systemic immune-inflammation index; *AFR*, albumin-to-fibrinogen ratio.

*p < 0.05; **p < 0.01; ***p < 0.001.

In the univariate Cox regression analysis for DFS, it was found that urothelial variants, T stage, LNM, nerve infiltration, LVI, hydronephrosis, and AFR were significant characteristics, listed in the univariate analysis section in **Table 4**. In the multivariate Cox regression analysis, LNM (HR = 5.954, 95%CI = 3.342–10.606, *p* < 0.001) and LVI (HR = 2.400, 95%CI = 1.349–4.270, *p* = 0.003) were identified as independent predictors for DFS (**Table 4**). The nomogram for predicting DFS is shown in **Figure 2c**.

**Table 4:** Univariate and Multivariate Cox regression analyses for the prediction of DFS in the training cohort (forward LR).

Indicators	Univariate analysis		Multivariate analysis	
	HR (95%CI)	*p*-value	HR (95%CI)	*p*-value
Urothelial variants				
No	Reference			
Yes	1.910 (1.140–3.199)	0.014^*^		
T stage				
T1, Ta, Tis	Reference			
T2	2.163 (1.134–4.126)	0.019^*^		
T3	4.457 (2.339–8.494)	<0.001^***^		
T4	9.651 (4.522–20.595)	<0.001^***^		
LNM				
No	Reference		Reference	
Yes	10.024 (6.233–16.122)	<0.001^***^	5.954 (3.342–10.606)	<0.001^***^
Nerve infiltration				
No	Reference			
Yes	4.139 (2.559–6.694)	<0.001^***^		
LVI				
No	Reference		Reference	
Yes	5.213 (3.257–8.343)	<0.001^***^	2.400 (1.349–4.270)	0.003^**^
Hydronephrosis				
No	Reference			
Yes	1.871 (1.140–3.072)	0.013^*^		
AFR				
≤13.24	Reference			
>13.24	0.416 (0.256–0.675)	<0.001^***^		

*HR*, hazard ratio; *CI*, confidence interval; *LNM*, lymph node metastasis; *LVI*, lymphovascular invasion; *AFR*, albumin-to-fibrinogen ratio.

*p < 0.05; **p < 0.01; ***p < 0.001.

### 3.3 Validation of the OS nomogram

**Figure 3:**
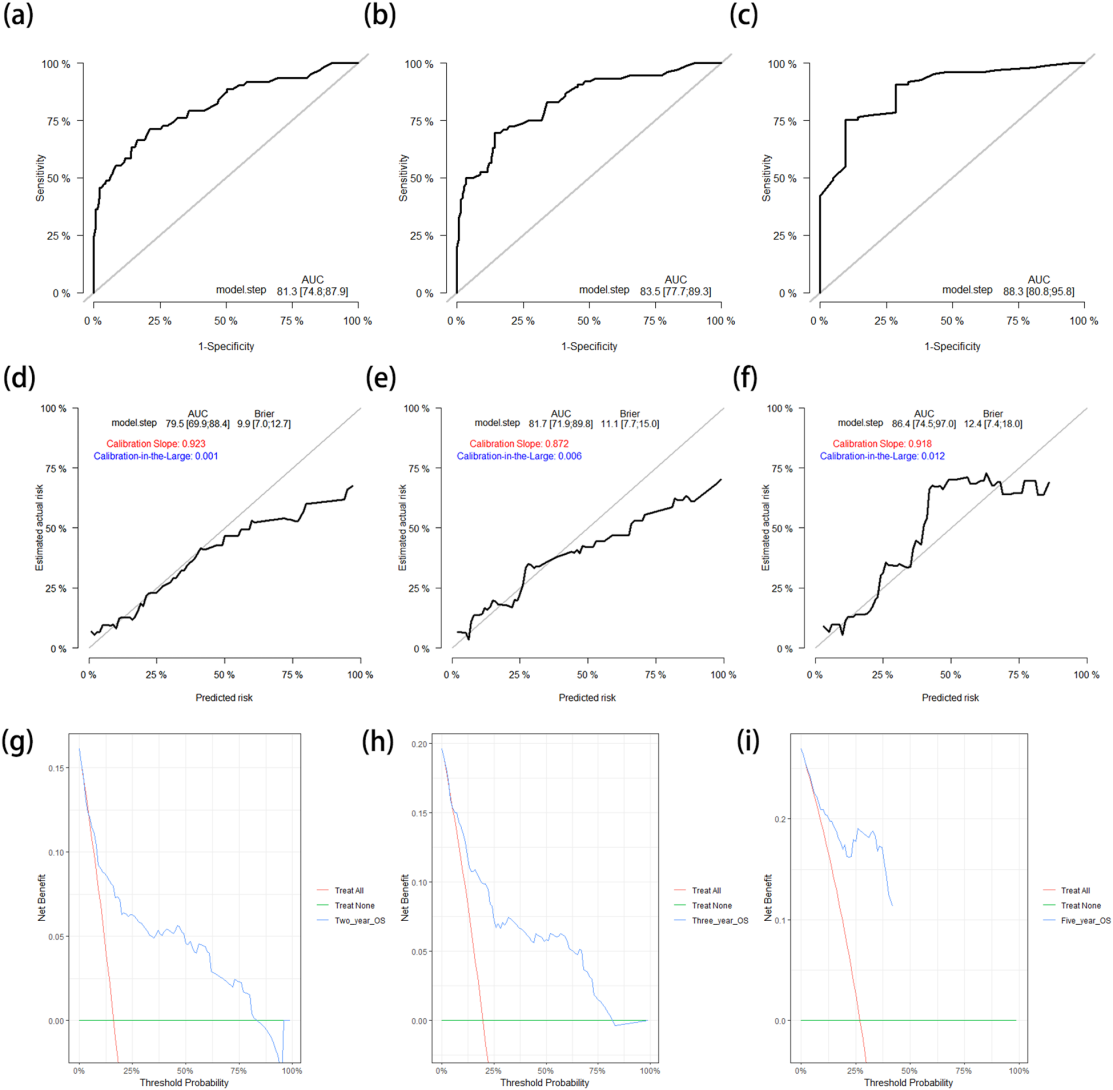
**(a–c)** ROC curves for predicting the 2-year **(a)**, 3-year **(b)**, and 5-year **(c)** OS rates in the training cohort. **(d–f)** Calibration curves with corrected AUCs and 95%CIs of the 2-year **(d)**, 3-year **(e)**, and 5-year **(f)** OS rates after 1,000 bootstrap resamplings in the training cohort. **(g–i)** DCA curves of the 2-year **(g)**, 3-year **(h)**, and 5-year **(i)** OS in the training cohort. *ROC*, receiver operating characteristic; *OS*, overall survival; *AUC*, area under the ROC curve; *CI*, confidence interval; *DCA*, decision curve analysis.

The C-index of the nomogram for the prediction of OS in the training cohort was 0.796 (95%CI = 0.741–0.850). The AUCs of the ROC curves for predicting the 2-, 3-, and 5-year OS rates in the training cohort were 0.813 (95%CI = 0.748–0.879), 0.835 (95%CI = 0.777–0.893), and 0.883 (95%CI = 0.808–0.958), respectively (**Figures 3a–c**). The calibration curves after 1,000 bootstrap resamplings are shown in **Figures 3d–f**. The corrected AUCs for the 2-, 3-, and 5-year OS rates were 0.795 (95%CI = 0.699–0.884), 0.817 (95%CI = 0.719–0.898), and 0.864 (95%CI = 0.745–0.970), respectively, with little change from the primary AUCs in the training cohort. The DCA curves for the 2-, 3-, and 5-year OS showed clinical benefits across a broad spectrum of threshold probabilities (**Figures 3g–i**).

**Figure 4:**
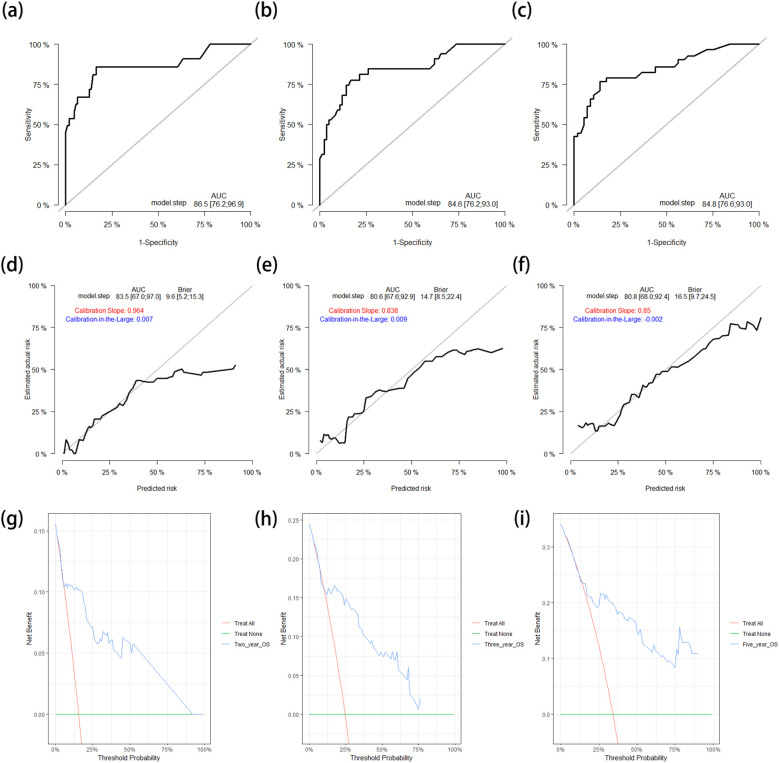
**(a–c)** ROC curves for predicting the 2-year **(a)**, 3-year **(b)**, and 5-year **(c)** OS rates in the test cohort. **(d–f)** Calibration curves with corrected AUCs and 95%CIs of the 2-year **(d)**, 3-year **(e)**, and 5-year **(f)** OS rates after 1,000 bootstrap resamplings in the test cohort. **(g–i)** DCA curves of the 2-year **(g)**, 3-year **(h)**, and 5-year **(g)** OS in the test cohort. *ROC*, receiver operating characteristic; *OS*, overall survival; *AUC*, area under the ROC curve; *CI*, confidence interval; *DCA*, decision curve analysis.

Subsequently, the OS nomogram model was applied to the test cohort. The *C*-index was 0.810 (95%CI = 0.743–0.877), which was similar to that in the training cohort. The ROC curves for predicting the 2-, 3-, and 5-year OS rates in the test cohort are shown in **Figures 4a–c**. The corresponding AUCs were 0.865 (95%CI = 0.762–0.969), 0.846 (95%CI = 0.762–0.930), and 0.848 (95%CI = 0.766–0.930), respectively, which are similar to the results in the training cohort. The calibration curves and the corrected AUCs with 1,000 bootstrap resamplings also showed little change from the AUCs in the test cohort (**Figures 4d–f**). The DCA curves for the 2-, 3-, and 5-year OS in the test cohort also showed clinical value (**Figures 4g–i**).

### 3.4 Validation of the CSS nomogram

**Figure 5:**
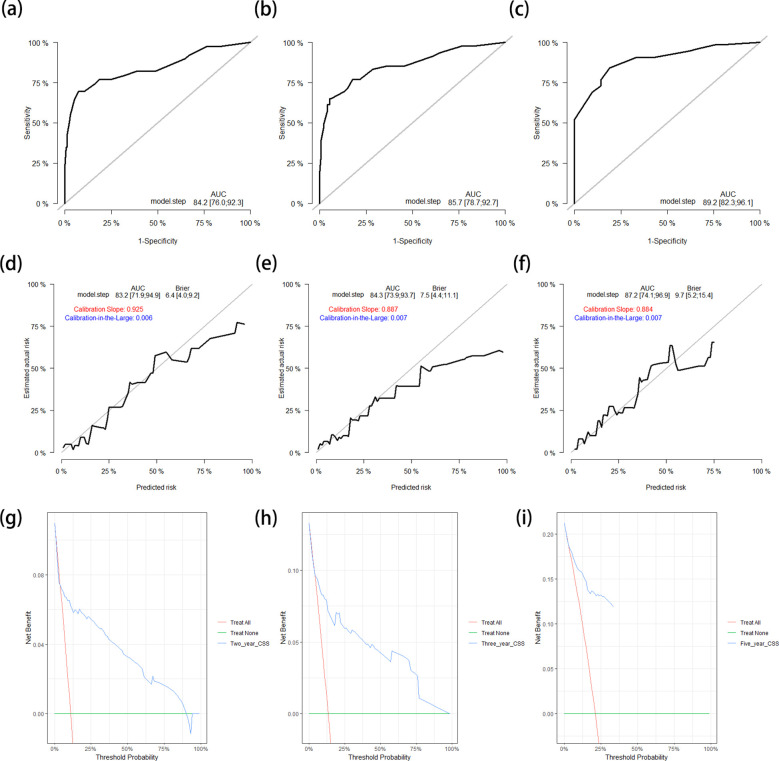
**(a–c)** ROC curves for predicting the 2-year **(a)**, 3-year **(b)**, and 5-year **(c)** CSS rates in the training cohort. **(d–f)** Calibration curves with corrected AUCs and 95%CIs of the 2-year **(d)**, 3-year **(e)**, and 5-year **(f)** CSS rates after 1,000 bootstrap resamplings in the training cohort. **(g–i)** DCA curves of the 2-year **(g)**, 3-year **(h)**, and 5-year **(i)** CSS in the training cohort. *ROC*, receiver operating characteristic; *CSS*, cancer-specific survival; *AUC*, area under the ROC curve; *CI*, confidence interval; *DCA*, decision curve analysis.

The *C*-index of the CSS nomogram in the training cohort was 0.836 (95%CI = 0.770–0.902). The AUCs of the ROC curves for predicting the 2-, 3-, and 5-year CSS rates in the training cohort were 0.842 (95%CI = 0.760–0.923), 0.857 (95%CI = 0.787–0.927), and 0.892 (95%CI = 0.823–0.961), respectively (**Figures 5a–c**). The calibration curves closely resembled the standard curve. Furthermore, the corrected AUCs also showed little change from the primary AUCs in the training cohort (with 2-, 3-, and 5-year CSS rates of 0.832, 0.843, and 0.872, respectively) (**Figures 5d–f**). These results demonstrate a good level of reproducibility for this model. The DCA curves for the 2-, 3-, and 5-year CSS in the training cohort showed that the model offered clinical benefits across a wide range of threshold probabilities (**Figures 5g–i**).

**Figure 6:**
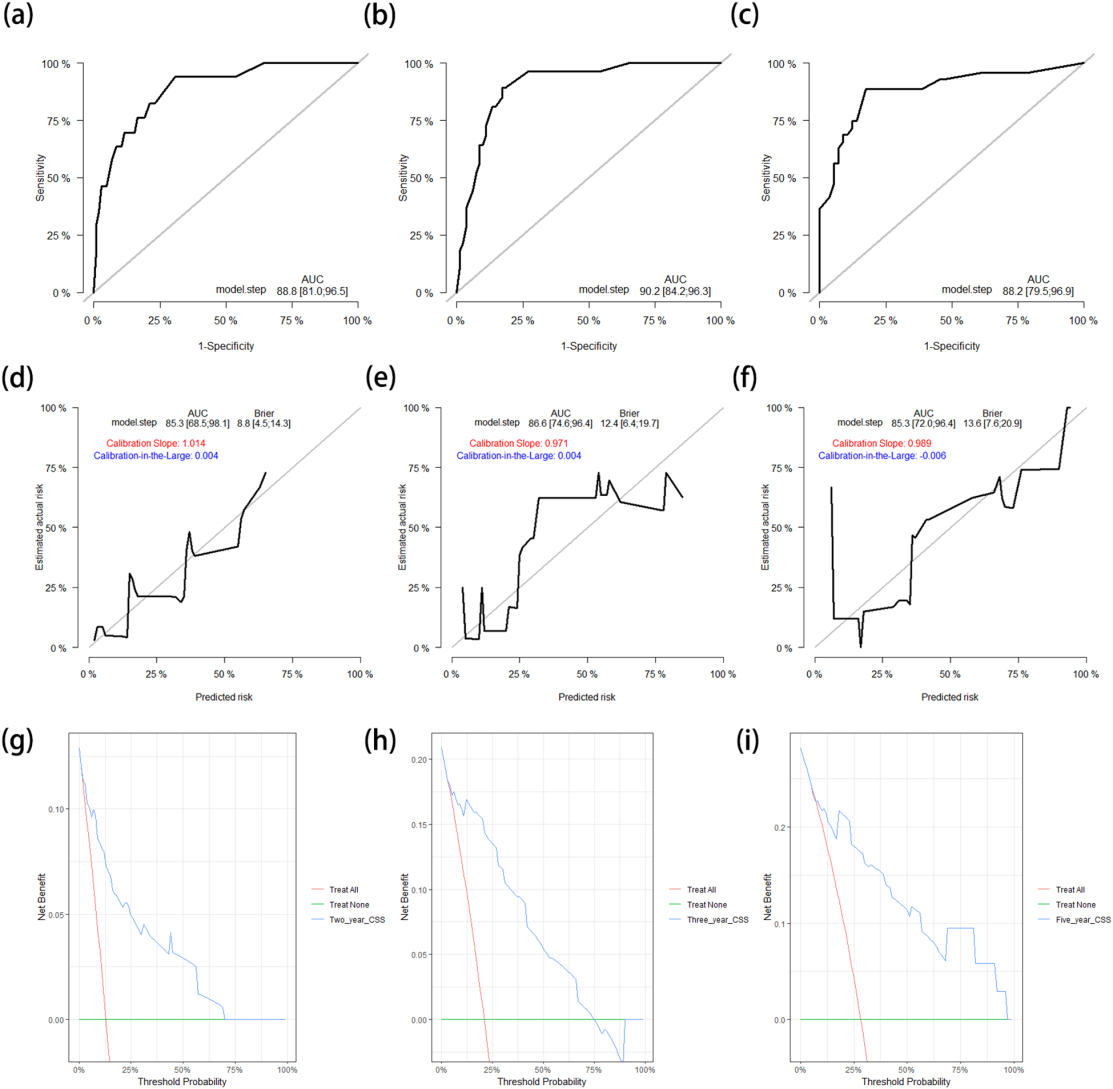
**(a–c)** ROC curves for predicting the 2-year **(a)**, 3-year **(b)**, and 5-year **(c)** CSS rates in the test cohort. **(d–f)** Calibration curves with corrected AUCs and 95%CIs of the 2-year **(d)**, 3-year **(e)**, and 5-year **(f)** CSS rates after 1,000 bootstrap resamplings in the test cohort. **(g–i)** DCA curves of the 2-year **(g)**, 3-year **(h)**, and 5-year **(i)** CSS in the test cohort. *ROC*, receiver operating characteristic; *CSS*, cancer-specific survival; *AUC*, area under the ROC curve; *CI*, confidence interval; *DCA*, decision curve analysis.

After applying the CSS nomogram model to the test cohort, the *C*-index was 0.842 (95%CI = 0.781–0.904), which was similar to that in the training cohort. The AUCs of the ROC curves for prediction of the 2-, 3-, and 5-year CSS rates in the test cohort were 0.888 (95%CI = 0.810–0.965), 0.902 (95%CI = 0.842–0.963), and 0.882 (95%CI = 0.795–0.969), respectively (**Figures 6a–c**). The calibration curves and the corrected AUCs changed little from the AUCs listed above (**Figures 6d–f**). The results of the DCA curves in the test cohort also showed value in clinical practice (**Figures 6g–i**).

### 3.5 Validation of the DFS nomogram

The *C*-index of the DFS nomogram in the training cohort was 0.762 (95%CI = 0.705–0.818). The ROC curves for prediction of the 2-, 3-, and 5-year DFS rates in the training cohort are shown in **Supplementary Figures S1A–C**. The corresponding AUCs were 0.777 (95%CI = 0.707–0.846), 0.782 (95%CI = 0.713–0.850), and 0.746 (95%CI = 0.659–0.833), respectively. Although the corrected AUCs after 1,000 bootstrap resamplings were similar to the primary AUCs, the calibration curves were not close to the standard curve (**Supplementary Figures S1D–F**). The DCA curves still showed useful clinical predictive value (**Supplementary Figures S1G, H**).

The DFS nomogram was applied to the test cohort, and the *C*-index [0.728 (95%CI = 0.658–0.797)] was close to the result in the training cohort. The AUCs of the ROC curves for predicting the 2-, 3-, and 5-year DFS rates in the test cohort also showed a high degree of differentiation compared with the training cohort (**Supplementary Figures S2A–C**). The corrected AUCs were also similar to the primary AUCs of the DFS nomogram in the test cohort. However, the calibration curves were still not close to the standard curve (**Supplementary Figures S2D–F**). The clinical application value was also confirmed in the DCA curves of the DFS nomogram in the test cohort (**Supplementary Figures S2G–I**).

## 4 Discussion

BUC, which ranges from NMIBC that easily recurs and commits patients to long-term invasive surveillance to MIBC with an inferior prognosis, is the ninth most common malignant tumor worldwide and contributes significantly to mortality and financial burden (20). Recently, comprehensive treatment protocols, including surgery, chemotherapy, radiotherapy, immunotherapy, and molecular targeted therapy, have significantly improved the prognosis of patients with BUC. Particularly for patients with MIBC and those with high or very high-risk NMIBC, RC with bilateral lymphadenectomy remains the standard curative treatment, providing accurate staging and adequate local and regional control (21, 22), which is also recommended by the guidelines of the European Association of Urology and the American Urological Association (23, 24). However, RC remains a complex surgical procedure with a considerable possibility of postoperative complications and a non-negligible perioperative mortality risk (24). Two retrospective studies demonstrated that nearly two-thirds of patients suffered complications within 90 days after RC, and mortality ranged from 1.5% to 2.0% 30 days postoperatively (25, 26). In an article based on large national databases and institutional series, the readmission rate was approximately 25% within 30 days of discharge (27). Therefore, there is an urgent need for effective in-hospital supervision and regular out-of-hospital screening for patients with BUC. Here, we propose a reliable prediction model based on common clinical characteristics to evaluate the survival probability of individual BUC patients after RC.

In this retrospective study, LNM and LVI were identified as independent risk factors for OS, CSS, and DFS in the multivariate analyses, which were then included in the three nomograms. Although tumor size was not independently associated with DFS, it was significantly related to the OS and CSS of patients with BUC after RC. Previous studies have demonstrated that tumor size, LNM, and LVI have substantial influence on higher TNM stages and the prognosis of BUC (16, 28–32), which is consistent with the results of our study. Indeed, age is the most significant single risk factor for the development of BUC and subsequent mortality upon diagnosis, particularly for assessment of the clinical and behavioral responses of patients with BUC after their associated therapies. Elderly patients encounter both clinical and systemic barriers that hinder access to appropriate treatment, and a satisfactory prognosis even after proper treatment (33, 34). It was demonstrated that age is a predictor of OS, with one study also providing proof that age could remarkably predict the OS of patients with high-grade BUC after RC (16). However, we did not find an independent relationship between age and CSS and DFS. A nomogram incorporating age could significantly predict the CSS of patients with BUC (31). Another study also provided proof that age is associated with the DFS and CSS of patients with BUC after RC (35). Although T stage has been proven to be independently related to OS (16, 32), CSS (31, 35), and DFS (35) in patients with BUC after RC, and a significant relationship was found between T stage and prognosis in the univariate analyses in this study, there was no significant relationship found in the multivariate analyses. This may be due to the limited sample size of the study.

In this study, preoperative AFR and urea nitrogen were demonstrated to be significantly associated with OS and CSS in patients with BUC after RC and were enrolled in the OS and CSS nomograms. Consistently, Biagio et al. demonstrated that preoperative AFR could serve as a predictor of the degree of malignancy and progression in patients with BUC (36). Moreover, three retrospective studies commonly determined preoperative AFR as an independent prognostic predictor of OS in patients with BUC undergoing RC (37–39). Claps et al. (37) also demonstrated the independent relationship between AFR and CSS. However, Li et al. (38) and Chen et al. (39) provided proof of the predictive value of AFR in DFS, which was not identified in our study. These incompletely consistent results may be explained by the different cutoff values for AFR. In addition, Pollack’s group found that urea nitrogen was significantly relevant to pelvic control of MIBC after RC (40). Moreover, we identified preoperative creatinine as an independent risk factor for OS, which was also proven in a prospective cohort study (41). Although preoperative DRR has been reported to be an independent predictor of prognosis in patients with BUC after RC (42, 43), we did not find a significant association in both univariate and multivariate analyses. In conclusion, the identified potential clinical laboratory biomarkers in our study could be convenient and cost-effective in helping urologists better monitor postoperative risk and make clinical decisions.

We innovatively used multidimensional clinical characteristics, including demographic, pathological, radiologic, and laboratory data, to develop nomograms for the prediction of OS, CSS, and DFS in patients with resectable BUC after RC. The OS and CSS nomograms showed high prediction accuracy in the *C*-indexes and ROC curves, reliability in the calibration curves with corrected AUCs, and clinical application value in the DCA curves in both cohorts. Furthermore, the patients from the Affiliated Hospital of Qingdao University were divided into two cohorts based on the diagnosis time, and the patients from Qingdao Campus of Qilu Hospital of Shandong University were combined into the test cohort in order to perform more convincing validation, which also supplemented the limitation of the sample size being too small to conduct complete external validation.

Despite successfully developing and validating nomograms to predict the individual survival probability of patients with BUC undergoing RC, our study has several limitations. First, the calibration curves of the DFS nomogram in both the training and test cohorts did not perform satisfactorily, which could be due to the limited sample size and follow-up time of the included patients. Second, due to the small number of patients who received neoadjuvant therapy and the lack of complete follow-up information, we did not analyze the potential impact of neoadjuvant or adjuvant therapy on the prognosis of patients with BUC after RC. However, several studies have proven survival benefits. Considering the limited number of patients in this study, high-quality, prospective research with a multicenter design and a large sample size should be conducted in the future.

## 5 Conclusion

This study focused on patients with resectable BUC receiving RC and identified age, tumor size, LNM, LVI, urea nitrogen, creatinine, and AFR as independent predictors for OS; tumor size, LNM, LVI, urea nitrogen, and AFR as independent predictors for CSS; and LNM with LVI as independent predictors for DFS. OS, CSS, and DFS nomograms were developed. These nomograms exhibited high accuracy, reliability, and clinical benefit in prediction in both cohorts.

## Data Availability

The raw data supporting the conclusions of this article will be made available by the authors, without undue reservation.
