# Supplementary material for: Development and validation of nomograms for predicting prognosis in patients with resectable bladder urothelial carcinoma undergoing radical cystectomy: a multicenter retrospective study
Source: Front Oncol. 2025 Jul 3;15:1571604. doi: 10.3389/fonc.2025.1571604 (PMC12268282; doi:10.3389/fonc.2025.1571604)
Supplement: Supplementary file 1 [file DataSheet1.docx]

**Supplementary Figure**


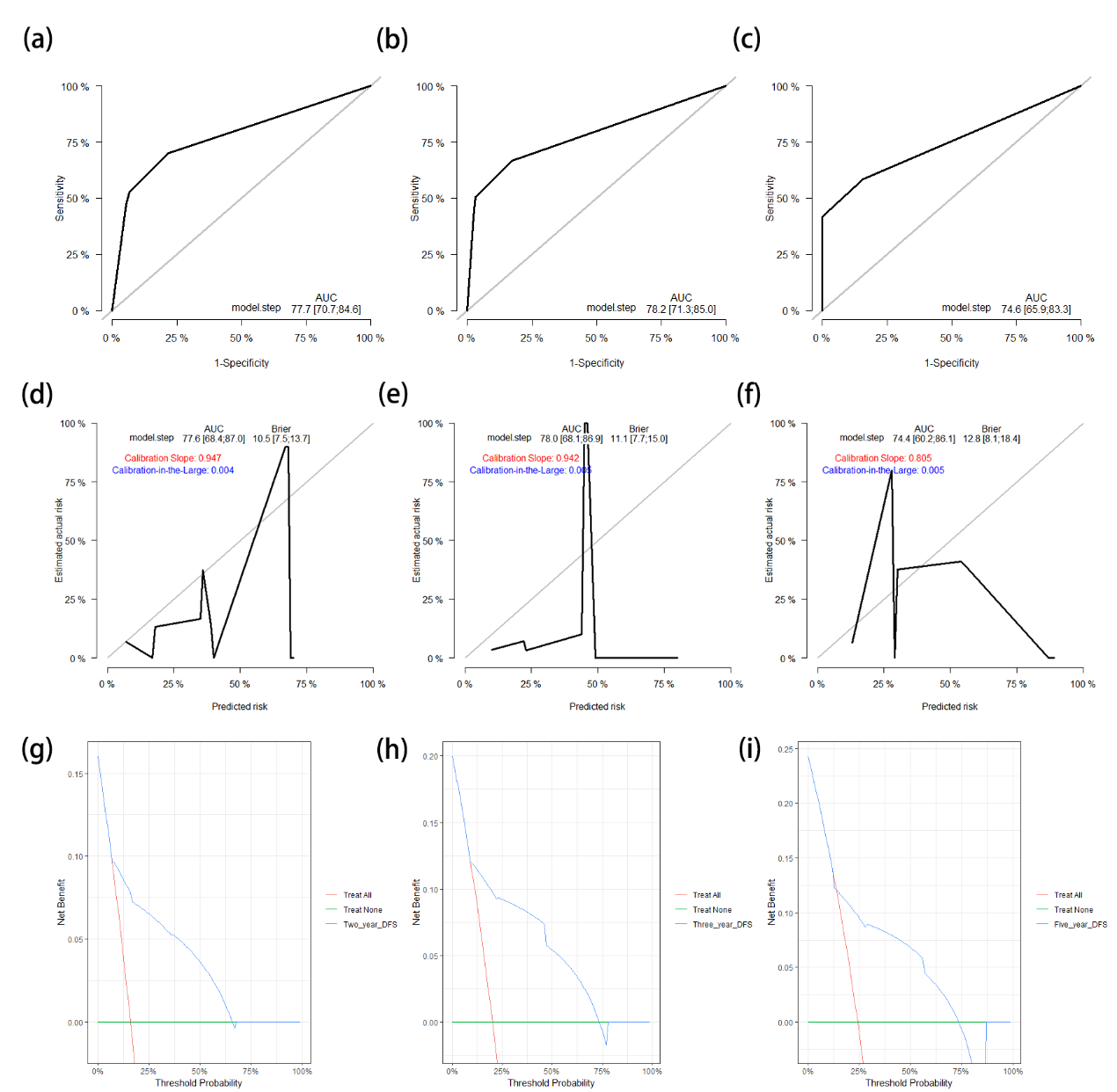


**Supplementary Figure. 1** The ROC curves for predicting 2-year DFS rate (a), 3-year DFS rate (b), and 5-year DFS rate (c) in the training cohort. The calibration curves with corrected AUCs and 95%CIs of 2-year DFS rate (d), 3-year DFS rate (e), and 5-year DFS rate (f) after 1000 Bootstrap resampling in the training cohort. The DCA curves of 2-year-DFS (g), 3-year-DFS (h), and 5-year-DFS (i) in the training cohort. ROC, receiver operating characteristic; DFS, disease-free survival; AUC, area under the ROC curve; CI, confidence interval; DCA, decision curve analysis.


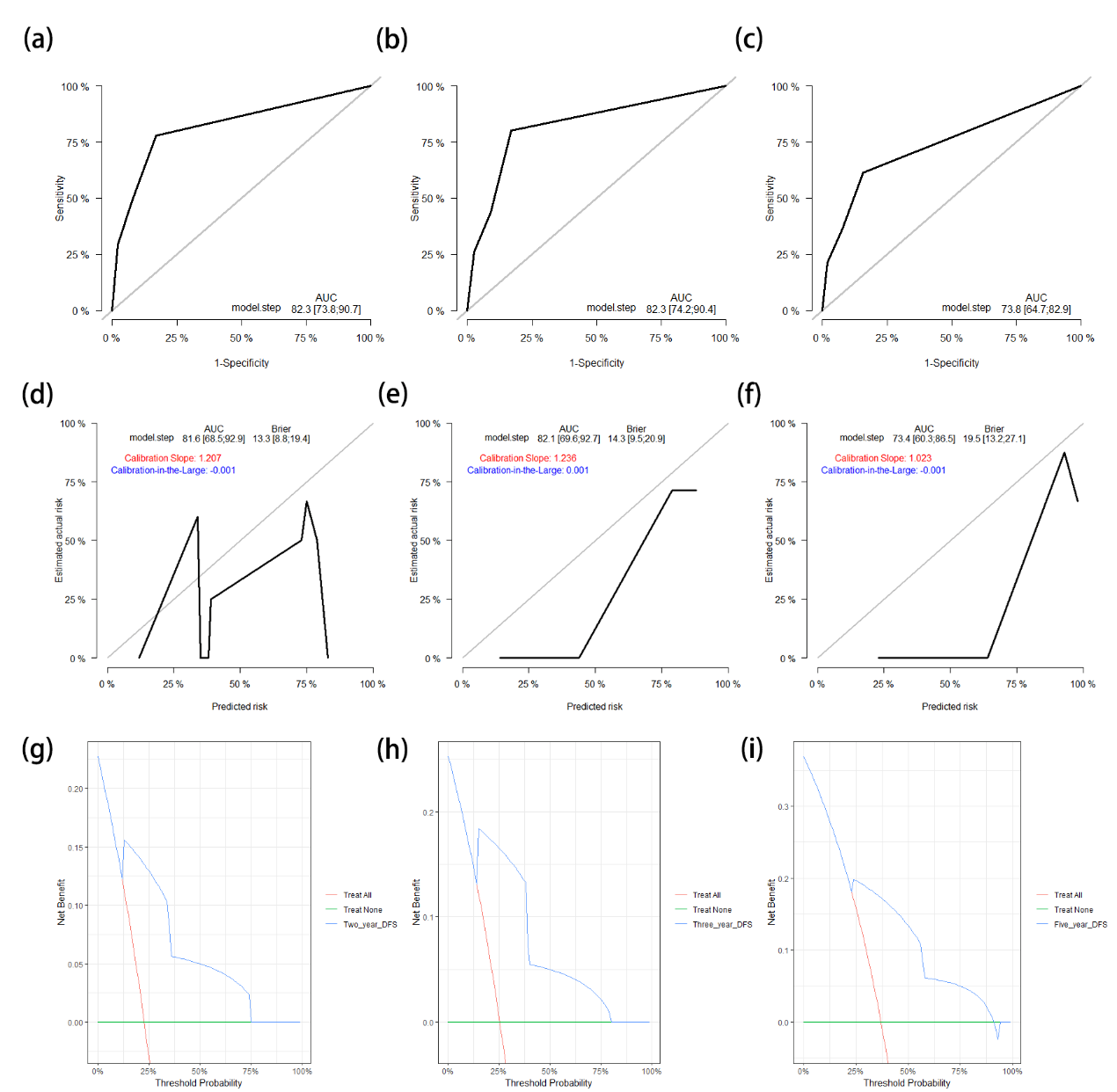


**Supplementary Figure. 2** The ROC curves for predicting 2-year DFS rate (a), 3-year DFS rate (b), and 5-year DFS rate (c) in the test cohort. The calibration curves with corrected AUCs and 95%CIs of 2-year DFS rate (d), 3-year DFS rate (e), and 5-year DFS rate (f) after 1000 Bootstrap resampling in the test cohort. The DCA curves of 2-year-DFS (g), 3-year-DFS (h), and 5-year-DFS (i) in the test cohort. ROC, receiver operating characteristic; DFS, disease-free survival; AUC, area under the ROC curve; CI, confidence interval; DCA, decision curve analysis.
