# Supplementary material for: Development and validation of nomograms for predicting prognosis in patients with resectable bladder urothelial carcinoma undergoing radical cystectomy: a multicenter retrospective study
Source: Front Oncol. 2025 Jul 3;15:1571604. doi: 10.3389/fonc.2025.1571604 (PMC12268282; doi:10.3389/fonc.2025.1571604)
Supplement: Supplementary file 2 [file Table1.docx]

| **Supplementary Table 1** Comparison of baseline characteristics between the two cohorts for CSS | | | | |
| --- | --- | --- | --- | --- |
| **Characteristics** | | **Training cohort (n=367)** | **Testing cohort (n=137)** | **P Value** |
| **Demography** | |  |  |  |
| **Age** | ≤66 | 193 (52.6%) | 73 (53.3%) | 0.889 |
|  | >66 | 174 (47.4%) | 64 (46.7%) |  |
| **Sex** | male | 320 (87.2%) | 116 (84.7%) | 0.461 |
|  | female | 47 (12.8%) | 21 (15.3%) |  |
| **BMI** | ≤23.9 | 189 (51.5%) | 68 (49.6%) | 0.710 |
|  | >23.9 | 178 (48.5%) | 69 (50.4%) |  |
| **Pathology** | |  |  |  |
| **Grade** | high grade | 324 (88.3%) | 119 (86.9%) | 0.663 |
|  | low grade | 43 (11.7%) | 18 (13.1%) |  |
| **Papillary** | yes | 151 (41.1%) | 66 (48.2%) | 0.156 |
|  | no | 216 (58.9%) | 71 (51.8%) |  |
| **Urothelial**  **Variants** | yes | 71 (19.3%) | 21 (15.3%) | 0.299 |
|  | no | 296 (80.7%) | 116 (84.7%) |  |
| **T stage** | T1 | 140 (38.1%) | 44 (32.1%) | 0.042^*^ |
|  | Ta | 13 (3.6%) | 4 (2.9%) |  |
|  | Tis | 4 (1.1%) | 2 (1.5%) |  |
|  | T2 | 111 (30.2%) | 44 (32.1%) |  |
|  | T3 | 76 (20.7%) | 22 (16.1%) |  |
|  | T4 | 23 (6.3%) | 21 (15.3%) |  |
| **Margin** | Positive | 8 (2.2%) | 10 (7.3%) | 0.013^*^ |
|  | Negative | 359 (97.8%) | 127 (92.7%) |  |
| **Tumor Size (cm)** | ≥4 | 143 (39.0%) | 57 (41.6%) | 0.590 |
|  | <4 | 224 (61.0%) | 80 (58.4%) |  |
| **LNM** | yes | 56 (15.3%) | 23 (16.8%) | 0.674 |
|  | no | 311 (84.7%) | 114 (83.2%) |  |
| BMI, body mass index; LNM, lymph node metastasis. *, P<0.05; **, P<0.01; ***, P<0.001. | | | | |

| **Supplementary Table 1** Continue | | | | |
| --- | --- | --- | --- | --- |
| **Characteristics** | | **Training cohort (n=367)** | **Testing cohort (n=137)** | **P Value** |
| **Nerve Infiltration** | yes | 64 (17.4%) | 27 (19.7%) | 0.556 |
|  | no | 303 (82.6%) | 110 (80.3%) |  |
| **LVI** | yes | 103 (28.1%) | 36 (26.3%) | 0.689 |
|  | no | 264 (71.9%) | 101 (73.7%) |  |
| **Imaging** | |  |  |  |
| **Hydronephrosis** | yes | 88 (24.0%) | 37 (27.0%) | 0.484 |
|  | no | 279 (76.0%) | 100 (73.0%) |  |
| **Laboratory** | |  |  |  |
| **Hemoglobin** | ≤139 | 186 (50.7%) | 75 (54.7%) | 0.417 |
|  | >139 | 181 (49.3%) | 62 (45.3%) |  |
| **Urea Nitrogen** | ≤6.34 | 185 (50.4%) | 74 (54.0%) | 0.471 |
|  | >6.34 | 182 (49.6%) | 63 (46.0%) |  |
| **Creatinine** | ≤79 | 192 (52.3%) | 69 (50.4%) | 0.697 |
|  | >79 | 175 (47.7%) | 68 (49.6%) |  |
| **NLR** | ≤2.19 | 190 (51.8%) | 79 (57.7%) | 0.238 |
|  | >2.19 | 177 (48.2%) | 58 (42.3%) |  |
| **PLR** | ≤130.29 | 185 (50.4%) | 84 (61.3%) | 0.029^*^ |
|  | >130.29 | 182 (49.6%) | 53 (38.7%) |  |
| **MLR** | ≤0.27 | 189 (51.5%) | 79 (57.7%) | 0.217 |
|  | >0.27 | 178 (48.5%) | 58 (42.3%) |  |
| **NPR** | ≤0.018 | 186 (50.7%) | 83 (60.6%) | 0.047^*^ |
|  | >0.018 | 181 (49.3%) | 54 (39.4%) |  |
| **SII** | ≤524.95 | 186 (50.7%) | 87 (63.5%) | 0.010^*^ |
|  | >524.95 | 181 (49.3%) | 50 (36.5%) |  |
| **DRR** | ≤1.05 | 188 (51.2%) | 67 (48.9%) | 0.643 |
|  | >1.05 | 179 (48.8%) | 70 (51.1%) |  |
| **AFR** | ≤13.24 | 180 (49.0%) | 68 (49.6%) | 0.906 |
|  | >13.24 | 187 (51.0%) | 69 (50.4%) |  |
| LVI, lymph-vascular invasion; NLR, neutrophil-to-lymphocyte ratio; PLR, platelet-to-lymphocyte ratio; MLR, monocyte-to-lymphocyte ratio; NPR, neutrophil-to-platelet ratio; SII, systemic immune inflammation index; DRR, de ritis ratio; AFR, albumin-to-ﬁbrinogen ratio; *, P<0.05; **, P<0.01; ***, P<0.001. | | | | |
