# Supplementary material for: Development and validation of nomograms for predicting prognosis in patients with resectable bladder urothelial carcinoma undergoing radical cystectomy: a multicenter retrospective study
Source: Front Oncol. 2025 Jul 3;15:1571604. doi: 10.3389/fonc.2025.1571604 (PMC12268282; doi:10.3389/fonc.2025.1571604)
Supplement: Supplementary file 3 [file Table2.docx]

| **Supplementary Table 2** Comparison of baseline characteristics between the two cohorts for DFS | | | | |
| --- | --- | --- | --- | --- |
| **Characteristics** | | **Training cohort (n=357)** | **Testing cohort (n=140)** | **P Value** |
| **Demography** | |  |  |  |
| **Age** | ≤66 | 191 (53.5%) | 76 (54.3%) | 0.875 |
|  | >66 | 166 (46.5%) | 64 (45.7%) |  |
| **Sex** | male | 310 (86.8%) | 119 (85.0%) | 0.592 |
|  | female | 47 (15.2%) | 21 (15.0%) |  |
| **BMI** | ≤23.9 | 180 (50.4%) | 69 (49.3%) | 0.820 |
|  | >23.9 | 177 (49.6%) | 71 (50.7%) |  |
| **Pathology** | |  |  |  |
| **Grade** | high grade | 315 (88.2%) | 122 (87.1%) | 0.737 |
|  | low grade | 42 (11.8%) | 18 (12.9%) |  |
| **Papillary** | yes | 149 (41.7%) | 69 (49.3%) | 0.127 |
|  | no | 208 (58.3%) | 71 (50.7%) |  |
| **Urothelial**  **Variants** | yes | 68 (19.0%) | 20 (14.3%) | 0.211 |
|  | no | 289 (81.0%) | 120 (85.7%) |  |
| **T stage** | T1 | 140 (39.2%) | 45 (32.1%) | 0.129 |
|  | Ta | 13 (3.6%) | 4 (2.9%) |  |
|  | Tis | 4 (1.1%) | 2 (1.4%) |  |
|  | T2 | 110 (30.8%) | 46 (32.9%) |  |
|  | T3 | 68 (19.1%) | 24 (17.1%) |  |
|  | T4 | 22 (6.2%) | 19 (13.6%) |  |
| **Margin** | Positive | 7 (2.0%) | 11 (7.9%) | 0.002^**^ |
|  | Negative | 350 (98.0%) | 129 (92.1%) |  |
| **Tumor Size (cm)** | ≥4 | 137 (38.4%) | 58 (41.4%) | 0.531 |
|  | <4 | 220 (61.6%) | 82 (58.6%) |  |
| **LNM** | yes | 50 (14.0%) | 23 (16.4%) | 0.492 |
|  | no | 307 (86.0%) | 117 (83.6%) |  |
| BMI, body mass index; LNM, lymph node metastasis. *, P<0.05; **, P<0.01; ***, P<0.001. | | | | |

| **Supplementary Table 2** Continue | | | | |
| --- | --- | --- | --- | --- |
| **Characteristics** | | **Training cohort (n=357)** | **Testing cohort (n=140)** | **P Value** |
| **Nerve Infiltration** | yes | 59 (16.5%) | 27 (19.3%) | 0.465 |
|  | no | 298 (83.5%) | 113 (80.7%) |  |
| **LVI** | yes | 95 (26.6%) | 33 (23.6%) | 0.486 |
|  | no | 262 (73.4%) | 107 (76.4%) |  |
| **Imaging** | |  |  |  |
| **Hydronephrosis** | yes | 81 (22.7%) | 40 (28.6%) | 0.169 |
|  | no | 276 (77.3%) | 100 (71.6%) |  |
| **Laboratory** | |  |  |  |
| **Hemoglobin** | ≤139 | 178 (49.9%) | 76 (54.3%) | 0.375 |
|  | >139 | 179 (50.1%) | 64 (45.7%) |  |
| **Urea Nitrogen** | ≤6.34 | 182 (51.0%) | 77 (55.0%) | 0.420 |
|  | >6.34 | 175 (49.0%) | 63 (45.0%) |  |
| **Creatinine** | ≤79 | 188 (52.7%) | 70 (50.0%) | 0.593 |
|  | >79 | 169 (47.3%) | 70 (50.0%) |  |
| **NLR** | ≤2.19 | 186 (52.1%) | 83 (59.3%) | 0.148 |
|  | >2.19 | 171 (47.9%) | 57 (40.7%) |  |
| **PLR** | ≤130.29 | 184 (51.5%) | 87 (62.1%) | 0.033^*^ |
|  | >130.29 | 173 (48.5%) | 53 (37.9%) |  |
| **MLR** | ≤0.27 | 188 (52.7%) | 81 (57.9%) | 0.296 |
|  | >0.27 | 169 (47.3%) | 59 (42.1%) |  |
| **NPR** | ≤0.018 | 181 (50.7%) | 85 (60.7%) | 0.044^*^ |
|  | >0.018 | 176 (49.3%) | 55 (39.3%) |  |
| **SII** | ≤524.95 | 184 (51.5%) | 91 (65.0%) | 0.007^**^ |
|  | >524.95 | 173 (48.5%) | 49 (35.0%) |  |
| **DRR** | ≤1.05 | 186 (52.1%) | 68 (48.6%) | 0.479 |
|  | >1.05 | 171 (47.9%) | 72 (51.4%) |  |
| **AFR** | ≤13.24 | 173 (48.5%) | 69 (49.3%) | 0.868 |
|  | >13.24 | 184 (51.5%) | 71 (50.7%) |  |
| LVI, lymph-vascular invasion; NLR, neutrophil-to-lymphocyte ratio; PLR, platelet-to-lymphocyte ratio; MLR, monocyte-to-lymphocyte ratio; NPR, neutrophil-to-platelet ratio; SII, systemic immune inflammation index; DRR, de ritis ratio; AFR, albumin-to-ﬁbrinogen ratio; *, P<0.05; **, P<0.01; ***, P<0.001. | | | | |
